# Supplementary material for: Acoustic and visual monitoring of Peale’s dolphins (Lagenorhynchus australis) in the Magellan Strait
Source: PeerJ. 2025 Jun 26;13:e19234. doi: 10.7717/peerj.19234 (PMC12248228; doi:10.7717/peerj.19234)
Supplement: Supplemental Information 1 [file peerj-13-19234-s001.pdf]

Acoustic and visual monitoring of Peale's Dolphins (*Lagenorhynchus Australis*) in the Magellan strait, supplementary materials

Franck Malige (1,2), Julie Patris (1,2), Benjamin Caceres (3), Jonathan Poblete (3), Hervé Glotin (1,2,4), Montserrat Saravía (3), Constanza Alarcon Vera (3), Valentin Barchasz (1,4), Valentin Gies (1,4), Sebastian Marzetti (1,4), Marjorie Fuentes-Riquelme (5,6), Diego Filún (7,8)

(1) Université de Toulon, Aix Marseille Univ, CNRS UMR 7020, LIS, Toulon, France
(2) CIAN, international center of artificial intelligence in natural acoustics
(3) Asociación de Investigadores del Museo de Historia Natural Río Seco, Chile
(4) SMIoT, university of Toulon, France
(5) NGO Centro de Estudios para la Conservación de Ecosistemas Marinos, Gorbea 2550, off 313, Santiago, Chile
(6) NGO Yaqu Pacha, Germany
(7) Alfred Wegener Institute, Germany
(8) Centro Ideal, universidad Austral de Chile, Chile

Contents

1 Material and methods **1**
1.1 Detection settings 1
1.2 Ethogram 2
1.3 Definition of clicks parameters 2

2 Results **3**
2.1 Clicks parameters 3

1 Material and methods

1.1 Detection settings

The dolphins click's detection strategy used in this paper contains three steps. First, we filter the recordings (high pass filter with cut frequency of 100 kHz, Butterworth filter of level 5) to get rid of all the sounds that we are not interested in (for example boat noise, low frequency noise from waves or wind). Secondly, we select the chunks of 1 ms for which the filtered signal is higher than a threshold defined as a multiple m_1 of the standard deviation of the whole filtered signal. This step selects all types of signal with high intensity above 100 kHz and, among them, the broadband sounds produced by shrimps or crabs, for example. Third, we calculate for each selected chunk, a spectrum thanks to a FFT of the signal (512 points, corresponding to 1ms). We then select only the chunks that have an energy between 120 and 140 kHz higher that m_2 times the energy between 30 and 90 kHz. m_2 defines another threshold. This step aims at rejecting the broadband signals corresponding to shrimps signals or knocks on the device. To adjust the detector and select the thresholds m_1 and m_2, we did a manual annotation of clicks in three files of the data set :

- in the file `20211208_140140UTC_V12.wav`, no NBHF clicks are present but there are many short broadband sounds and a low frequency background noise.
- in the file `20211209_141357UTC_V12.wav` there are 328 NBHF clicks (with usually low SNR) and many short broadband sounds,
- in the file `20211210_054551UTC_V12.wav` there are 1477 NBHF clicks, some with a very good SNR.

Then we passed the detector varying the different thresholds m_1 and m_2 and build the true positive rate (TPR, or Precision) versus false negative rate (FNR, which is also 1-Recall) curve, based on these three files. For each value of m_1 and m_2, we computed the rate of missed clicks (FNR, abscissa) and the rate of real dectections (TPR or Precision, ordinate). The results are presented in the figure 1.

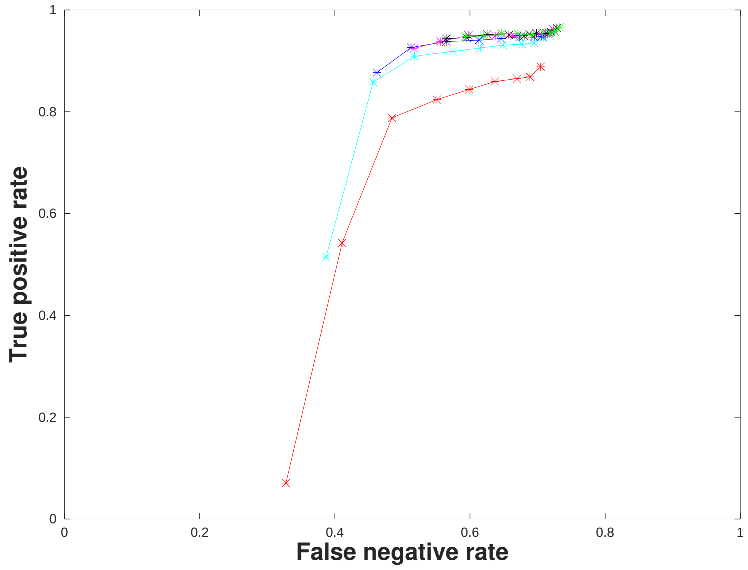

Figure 1: Decision curves, for different values of m_1 and m_2. Each curve is for one value of m_2 (m_2=0.5 / 0.75 / 1 / 1.25 / 1.5 / 1.75 : red, light blue, blue, violet, black, green) and the dots on each curve correpond to the values 4, 5, 6, 7, 8, 9, 10, 11, 12 for m_1 (from left to right). In absissa, the false negative rate and in ordinate the true positive rate.

With these results we selected the thresholds $m_1 = 6$ and $m_2 = 1.25$, taking into account that the computation time is much longer if the value of m_1 is lower. Furthermore, these values are the same as those used in the Patris et al. (2023) study, where the same device was used. With these values, the detector is not very sensitive, but it is rather precise: we estimate to have a percentage of false negatives around 60% and a percentage of true positives around 95%. That is to say that we lose a large number of real clicks but the detections corresponds to real dolphin clicks at 95%.

1.2 Ethogram

We defined four behaviors for Peale's dolphins in our study :

- Slow travel : Defined as a group of Peale's dolphins moving in a consistent direction (typically North to South or South to North) at a moderate speed. Moderate speed is operationally defined as instances where individuals surface more than twice during their transit across the bay.
- Fast travel : Characterized by directional movement at a higher velocity, with individuals surfacing only once or twice while crossing the bay. Directionality remains constant, typically along a North-South or South-North axis.
- Foraging : Identified when a group exhibits frequent changes in direction while maintaining a moderate speed. This behavior is also associated with prolonged dives lasting more than 20-30 seconds, possibly indicating prey searching or capture attempts.
- Social interactions : Encompasses non-travel behaviors, including tail fluke smacking, aerial behaviors such as breaching or jumping, and rapid accelerations, which may indicate social play or agonistic interactions.

1.3 Definition of clicks parameters

Consider an acoustic signal $s(t)$, and its Fourier transform, $S(f) = \int_{-\infty}^{+\infty} s(t)e^{-2i\pi ft}dt$. The following parameters are classical in the studies involving Narrow Band High Frequency (NBHF) species (Au 1993).

- The energy of the signal $E = \int_{-\infty}^{+\infty} |s(t)|^2 dt \ = \int_{-\infty}^{+\infty} |S(f)|^2 df$
- The amplitude of the signal, which is the maximum value of the signal s
- The amplitude peak-to-peak of the signal, which is the maximum value minus the minimum value of s
- The mean date of the signal defined by $t_c = \frac{\int_{-\infty}^{+\infty} t|s(t)|^2 dt}{\int_{-\infty}^{+\infty} |s(t)|^2 dt}$.
- The duration 'rms' : $\Delta t = \sqrt{\frac{\int_{-\infty}^{+\infty} (t-t_c)^2|s(t)|^2 dt}{\int_{-\infty}^{+\infty} |s(t)|^2 dt}}$.
- The duration at -10 dB ($\Delta t_{\ (-10dB)}$) is defined as the duration when the envelope of the signal is above the maximum of the envelope divided by $\sqrt{10}$. In practice, we get the envelope taking the modulus of the Hilbert transform of the signal.
- The duration at -20 dB ($\Delta t_{\ (-20dB)}$) is defined as the duration when the envelope of the signal is above the maximum of the envelope divided by 10
- The peak frequency f_p of the signal is the frequency where the spectrum $|S(f)|$ of the signal is maximum.
- The 'centroid' frequency f_c is defined by the formula $f_c = \frac{\int_{-\infty}^{+\infty} f|S(f)|^2 df}{\int_{-\infty}^{+\infty} |S(f)|^2 df}$.
- The 'rms' bandwidth : $\Delta f = \sqrt{\frac{\int_{-\infty}^{+\infty} (f-f_c)^2|S(f)|^2 df}{\int_{-\infty}^{+\infty} |S(f)|^2 df}}$
- The bandwidth at -3 dB ($\Delta f_{\ (-3dB)}$) is the length of the interval around f_p where the modulus of the spectrum is larger than $\frac{max(|S(f)|)}{\sqrt{2}}$ (it is equivalent to $max(|S(f)|^2 - 3\text{dB}$ when $max(|S(f)|^2$ is in dB).
- The bandwidth at -10 dB ($\Delta f_{\ (-10dB)}$) is the length of the interval around f_p where the modulus of the spectrum is larger $\frac{max(|S(f)|^2)}{\sqrt{10}}$.
- The quality factor at -3 dB is $Q_{\ (3dB)} = \frac{f_c}{\Delta f_{(-3dB)}}$.
- The quality factor 'rms' is $Q_{rms} = \frac{f_c}{\Delta f}$.
- The duration-bandwidth product 'rms' is $\Delta t \times \Delta f = \sqrt{\frac{\int_{-\infty}^{+\infty} (t-t_c)^2|s(t)|^2 dt}{\int_{-\infty}^{+\infty} |s(t)|^2 dt}} \times \sqrt{\frac{\int_{-\infty}^{+\infty} (f-f_c)^2|S(f)|^2 df}{\int_{-\infty}^{+\infty} |S(f)|^2 df}}$

These definitions can be found in Au (1993).

2 Results

2.1 Clicks parameters

Statistical distributions of the parameters measured for all the detections of the HighBlue device, along with the statistical distribution of the parameters of the subset of visually confirmed and "on-axis" clicks are presented in figure 2.

References

Au, W. (1993). *The sonar of dolphin.* Springer New York, NY.

Patris, J., Malige, F., Hamame, M., Glotin, H., Barchasz, V., Gies, V., Marzetti, S., and Buchan, S. (2023). Medium-term acoustic monitoring of small cetaceans in patagonia, chile. *PeerJ*, 11:e15292.

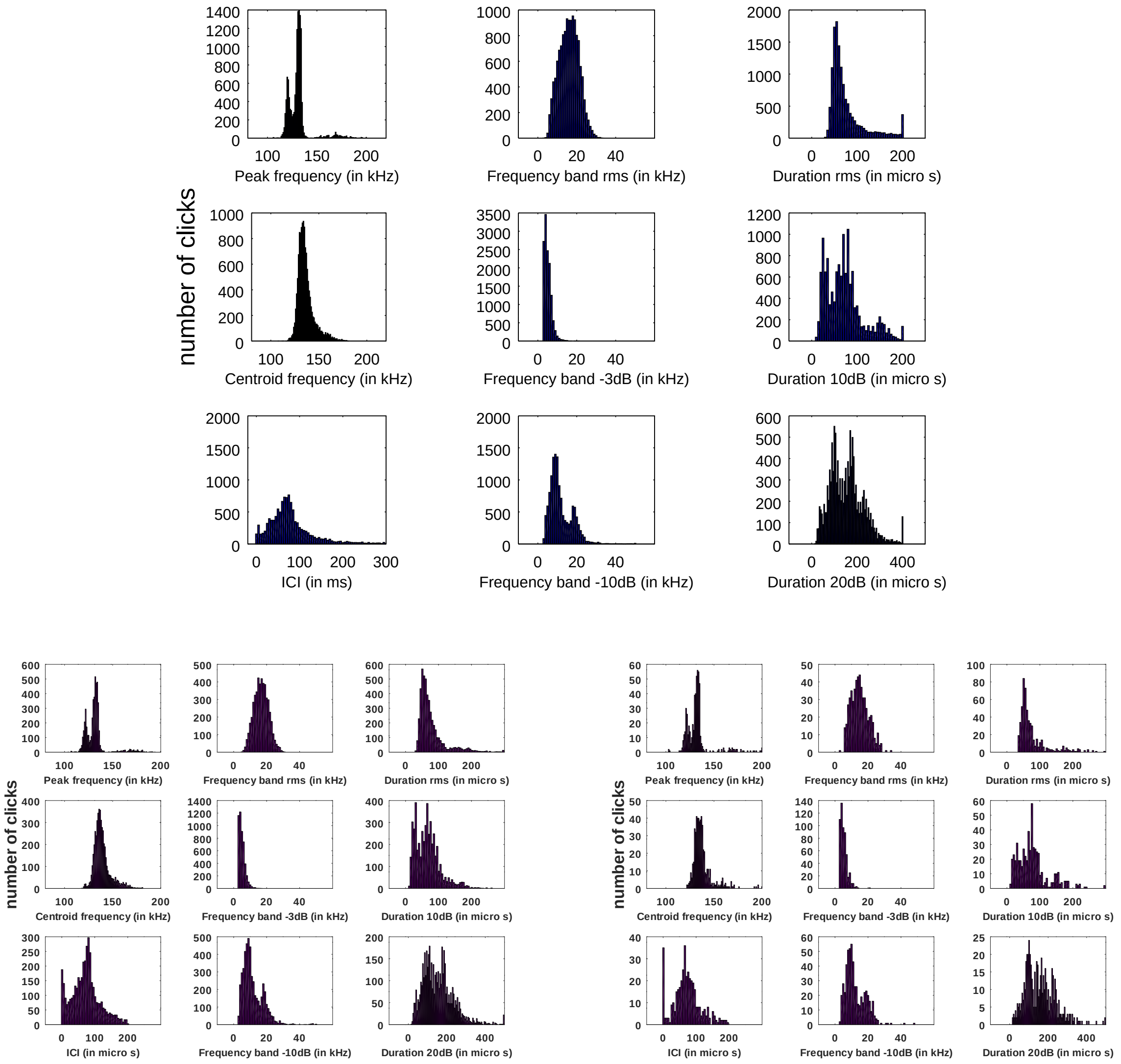

Figure 2: <u>Top</u> : histograms of click parameters : peak frequency, centroid frequency, inter-click interval (ICI), 'rms' bandwidth, '-3 dB' bandwidth, '-10 dB' bandwidth, 'rms' duration, '-10 dB' duration and '-20 dB' duration. <u>Bottom left</u> : The same histograms for clicks with a visual counterpart <u>Bottom right</u> : The same histograms for "on -axis" clicks.
